# Addressing challenges in pediatric thrombosis: a comprehensive guideline development

**DOI:** 10.3389/fped.2025.1519517

**Published:** 2025-01-27

**Authors:** Yin Wang, Qinan Yin, Jiangting Liao, Na Wang, Li Li, Siyan Li, Qingxia Zhang, Feifei Yu, Jianchun Luo, Hongmei Wang, Die Hu, Wenyi Li, Biao Li, Jingjing Chen, Limei Dong, Min Luo, Yu Yan, Lie Dong, Zheng Ding, Xi Wei, Jiadan Yang, Shenglan Tan, Lian Li, Xi Zheng, Liuyun Wu, Yong Yang, Linan Zeng, Jinqi Li, Lizhu Han, Yuan Bian

**Affiliations:** ^1^ Department of Pharmacy, Personalized Drug Therapy Key Laboratory of Sichuan Province, Sichuan Academy of Medical Sciences & Sichuan Provincial People's Hospital, School of Medicine, University of Electronic Science and Technology of China, Chengdu, China; ^2^ Department of Pharmacy, The Second Affiliated Hospital of Chongqing Medical University, Chongqing, China; ^3^ Department of Pharmacy, Guizhou Provincial People Hospital, Guiyang, Guizhou, China; ^4^ School of Pharmaceutical Sciences, Capital Medical University, Beijing, China; ^5^ Department of Pharmacy, Fengtai District Maternal and Child Health Care Hospital, Beijing, China; ^6^ Department of Pharmaceutical, National Clinical Research Center for Child Health and Disorders, Ministry of Education Key Laboratory of Child Development and Disorders, Chongqing Key Laboratory of Pediatrics, Children’s Hospital of Chongqing Medical University, Chongqing, China; ^7^ Department of Pharmacy, The Affiliated Hospital, Southwest Medical University, Luzhou, China; ^8^ Department of Pharmacy, The First Affiliated Hospital of Chongqing Medical University, Chongqing, China; ^9^ Department of Pharmacy, Affiliated Hospital of Southwest Jiaotong University, The Third People’s Hospital of Chengdu, Chengdu, Sichuan, China; ^10^ Department of Pharmacy, Qianwei County People’s Hospital, Leshan, Sichuan, China; ^11^ Department of Pharmacy, The People’s Hospital of Leshan, Leshan, Sichuan, China; ^12^ Department of Pharmacy/Evidence-Based Pharmacy Center, West China Second University Hospital, Sichuan University, Chengdu, China; ^13^ Department of Pharmacy, The Affiliated Hospital of Southwest Medical University, Luzhou, Sichuan, China; ^14^ Department of Pharmacy, West China Hospital, Sichuan University, Chengdu, China; ^15^ Department of Pharmacy, Fuwai Hospital, Chinese Academy of Medical Sciences, Beijing, China; ^16^ Department of Pharmacy, Third Affiliated Hospital of Naval Medical University, Shanghai, China; ^17^ Department of Pharmacy, Second Xiangya Hospital of Central South University, Changsha, Hunan, China; ^18^ Department of Pharmacy, The Fourth People’s Hospital of Chengdu, Chengdu, China

**Keywords:** pediatric thrombosis, rational drug use, thrombosis prevention and treatment, pharmaceutical practice guideline, anticoagulation

## Abstract

**Background:**

Pediatric thrombosis is a relatively rare but severe condition in the field of pediatrics, with far-reaching consequences. Recent studies have indicated a rising incidence of this disease in children over the years. Additionally, the pharmacological treatment of thrombotic diseases in children faces numerous challenges. Due to significant physiological differences between children and adults, guidelines for the prevention and treatment of thrombotic diseases in adults cannot be directly applied to pediatric patients.

**Purpose:**

A systematic review of the existing evidence-based medical literature should be conducted to propose pharmacological prevention and treatment recommendations for pediatric thrombotic diseases. Developing a comprehensive and practical pharmacotherapy guideline for the prevention and treatment of pediatric thrombotic diseases is essential to enhancing the rational use of medications in managing these conditions in children.

**Methods:**

The guideline development followed the World Health Organization's (WHO) Handbook for Guideline Development. This involves systematically searching and extensively collecting data on common medication issues in the prevention and treatment of pediatric thrombosis nationwide. The Delphi method was used to survey experts and identify the clinical issues to be included. Subsequently, a systematic literature review was conducted to evaluate existing primary studies, systematic reviews, and guidelines or consensus statements from professional organizations. The quality of the evidence was assessed using the Grading of Recommendations Assessment, Development and Evaluation (GRADE) approach. The Delphi method was employed again to reach a consensus on the recommendations and evidence levels. This process was culminated in the development of the Guideline for Pharmacological Management of Thrombotic Diseases in Children.

**Results:**

During the guideline development process, a total of 29 clinical issues were collected and evaluated by 78 experts in clinical pharmacy and clinical medicine. Through two rounds of surveys, 13 clinical issues were selected. Under the supervision of two methodologists, 13 clinical pharmacotherapy recommendations were formulated.

**Conclusion:**

By conducting a comprehensive assessment of the feasibility and safety of clinical practices, the guideline provides specific anticoagulant medication recommendations for pediatric healthcare professionals. This will help enhance the prevention and treatment of pediatric thrombosis, promoting more standardized and effective medical practices.

## 1 Introduction

The United Nations Convention on the Rights of the Child defines a child as an individual under the age of 18. Children are categorized into six stages: neonatal period (0–28 days), infancy (28 days–1 year), toddlerhood (1–3 years), preschool age (3–6 years), school age (6–12 years), and adolescence (12–18 years). Pediatric thrombosis, although relatively rare, is a disease with far-reaching consequences and has garnered widespread attention in the medical community over the past few decades. The incidence of venous thromboembolism (VTE) in children is approximately 0.000007%–0.000014% (1–5). It is noteworthy that in hospitalized children, the presence of risk factors such as central venous catheters (CVC), surgical history, malignant tumors, severe infections, severe burns or trauma, medications, and immobility during hospitalization can increase the incidence of VTE to 0.58% (6). Compared to VTE, arterial thromboembolism (ATE) in children is less common, with an average annual incidence of 0.24% (7–9).

Conducting clinical drug research in children is often challenging due to ethical issues, resulting in a limited number of anticoagulant medications approved for pediatric use (10). There are significant differences between adults and children in the epidemiology and pathophysiology of thrombosis, as well as in the pharmacodynamics and pharmacokinetics of antithrombotic drugs. Additionally, many pediatric antithrombotic treatment protocols are derived from adult practices, such as the use of direct oral anticoagulants (DOAC) in children, for which evidence is extrapolated from adult data (11). Therefore, there is an urgent need to develop pharmaceutical practice guidelines for the prevention and treatment of pediatric thrombosis to establish rational and standardized treatment protocols for anticoagulant medications in children at the current stage.

The Guideline for Pharmacological Management of Thrombotic Diseases in Children (hereafter referred to as the guideline) aim to provide a comprehensive and systematic reference for clinical practitioners, researchers, and decision-makers based on a synthesis of current evidence in evidence-based medicine. The guideline seeks to enhance understanding of pediatric thrombosis and facilitate the formulation of more effective pharmacological treatment decisions. The guideline will address specific thrombotic events in children, such as intracranial venous and arterial thrombosis, CVC-related venous thrombosis, etc. They will also explore thromboprophylaxis strategies during perioperative periods (e.g., cardiac surgery, orthopedic surgery) and for specific disease states that predispose to thrombosis [e.g., purpura fulminans (PF), inflammatory bowel disease (IBD), systemic lupus erythematosus (SLE)]. Furthermore, the guideline will present detailed recommendations for the prevention and treatment of thrombosis in children, aiming to provide practical guidance for managing these conditions effectively.

## 2 Methodology

The guideline was initiated and developed under the leadership of the Chinese Medical Association, Chinese Society of Clinical Pharmacy and the Chinese Society of Cardiothoracic and Vascular Anesthesiology, Cardiovascular Pharmacy Division. The expert panel members were initially established on January 28, 2023, and the official launch occurred on February 6, 2023. The revision process spanned 16 months, concluding with final revisions. The Chinese Medical Association, Chinese Society of Clinical Pharmacy and the Chinese Society of Cardiothoracic and Vascular Anesthesiology, Cardiovascular Pharmacy Division took responsibility for initiating and developing the guideline, while the Evidence-Based Medicine Center of West China Hospital provided methodological and evidence-based support.

**Figure 1:**
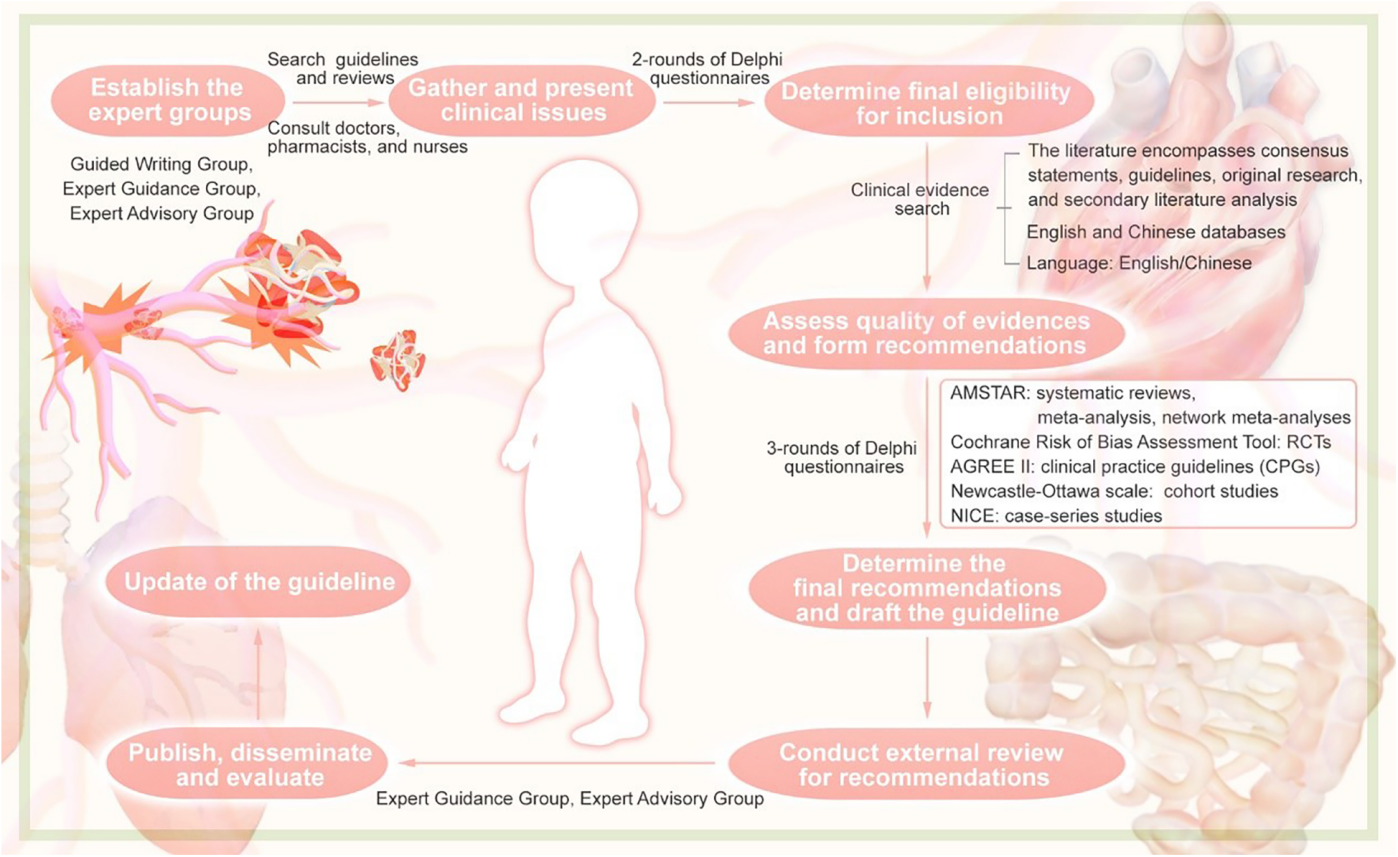
Flowchart of the guideline.

The guideline were developed following the guideline development process outlined in the WHO Handbook for Guideline Development (2014) (12). This guideline conforms to the requirements of the Appraisal of Guidelines for Research and Evaluation (AGREE) II (13) tool and adheres to the standards of the Reporting Items for Practice Guidelines in Healthcare (RIGHT) (14) checklist. The guideline have been registered on the Practice Guideline Registration for Transparency (PREPARE) platform (registration number: PREPARE-2022 CN758). The overall process for the formation of the guideline can be taken from Figure 1.

### 2.1 Scope and target audience of the guideline

The guideline primarily targets children under 18 years old with thrombotic diseases. Healthcare providers, including doctors, nurses, and pharmacists, as well as patients and their caregivers, who are involved in providing medical care, can benefit from this guideline.

### 2.2 Proposal and identification of clinical issues

The Guideline Writing Group, through the review of relevant guidelines and systematic reviews, initially identified 20 clinical issues. Subsequently, two rounds of questionnaires were conducted using the Delphi method. In the first round, experts rated each of the initial 20 clinical issues based on their perceived importance using a scale of 1–5 through an online questionnaire. For the issues with a coefficient of variation ≥25% and for the nine additional clinical issues suggested by the experts, a second round of rating was conducted. Across both rounds of surveys for the guideline, a total of 104 questionnaires were collected from 37 medical institutions in 21 provinces (autonomous regions, municipalities directly under the Central Government). Based on the importance ratings assigned to each clinical issue, a final set of 13 clinical issues were ultimately included in the guideline.

### 2.3 Evidence retrieval, assessment, and grading

For the final set of clinical issues included in the guideline, two members of the writing group conducted comprehensive searches across multiple Chinese and English databases using the Population, Intervention, Comparison, and Outcome (PICO) framework. The population (P) of interest was children under 18 years of age. The intervention (I) was intravenous (IV) dexmedetomidine in any dose and for any duration. The comparators (C) were with or without any antithrombotic drug. The outcomes (O) selected and prioritised as critical by the panel were: (1) major bleeding, (2) clinically relevant nonmajor bleeding, (3) any other adverse event, (4) mortality, (5) thrombus regression or recurrence, and (6) quality of life. The databases searched included Medline, Embase, Cochrane Library, Web of Science, UpToDate, ClinicalTrials.org, CBM (China Biology Medicine disc), China National Knowledge Infrastructure (CNKI), Chinese Biomedical Literature Database (CBM), Chinese Journal Full-text Database (CNKI), VIP, and Wanfang Data. Commonly used foreign clinical guideline websites included the National Guideline Clearinghouse (NGC), the Guideline International Network (GIN), the Scottish Intercollegiate Guidelines Network (SIGN), the National Institute for Health and Care Excellence (NICE), and the WHO website. The search covered literature indexed from database inception to March 31, 2023, in both Chinese and English languages. The search strategies involved using titles, keywords, or abstracts and were tailored to each database. The retrieved literature included systematic reviews, meta-analyses, network meta-analyses, original studies [such as randomized controlled trials (RCTs), cohort studies, and case-control studies], and clinical guidelines.

The guideline used the Assessment of Multiple Systematic Reviews (AMSTAR) (15) tool to evaluate the methodological quality of systematic reviews, meta-analyses, and network meta-analyses. The Cochrane Risk of Bias Assessment Tool (16) was employed for evaluating RCTs. AGREE II (13) was utilized to assess the methodological quality of the guidelines themselves. The Newcastle-Ottawa Scale (17) was applied for evaluating cohort studies, and the NICE UK Evaluation Tool (18) were used for assessing case-series studies. The evaluation was conducted independently by two reviewers, with discrepancies resolved through consensus or consultation with a third party. The quality of the evidence was graded using the GRADE approach (19), categorized into High, Moderate, Low, and Very Low. Recommendation strength was determined according to Evidence retrieval and evaluation in evidence-based medicine (20), distinguishing between strong and weak recommendations (Table 1).

**Table 1:** Definition of evidence quality classification and definition of recommendation strength.

Classification	Definition
Evidence quality	
High	We are very confident that the true effect lies close to that of the estimate of the effect.
Moderate	We are moderately confident in the effect estimate: The true effect is likely to be close to the estimate of the effect, but there is a possibility that it is substantially different.
Low	Our confidence in the effect estimate is limited: The true effect may be substantially different from the estimate of the effect.
Very Low	We have very little confidence in the effect estimate: The true effect is likely to be substantially different from the estimate of effect.
Recommendation strength	
Strong	Consider a recommendation as strong if it is supported by high-quality evidence, aligns closely with clearly defined values and preferences, and incurs minimal costs and resource expenditures.
Weak	Consider a recommendation as weak if there is significant uncertainty regarding the quality of evidence, alignment with values and preferences, and associated costs and resource expenditures.

### 2.4 Formation of guideline recommendations

The Guideline Writing Group initially drafted recommendations for the prevention and treatment of pediatric thrombosis with antithrombotic drugs through the first two rounds of Delphi questionnaires, based on domestic and international evidence and considering the costs and benefits of interventions. In May, July, and December 2023, a third round of Delphi surveys was conducted to reach consensus on the recommendations and evidence levels for the clinical issues. In April 2024, following expert group discussions, recommendations with an agreement rate of ≥90% among experts were defined as the guideline recommendations. After external review and revisions, 13 final guideline recommendations were formulated.

### 2.5 External review of guideline recommendations

The consensus recommendations were submitted to the Expert Advisory Group for review. The Guideline Writing Group discussed the feedback, made revisions to the recommendations accordingly, and then submitted the revised recommendations to the Expert Guidance Group for approval, finalizing the recommendations.

### 2.6 Writing and updating of the guideline

After approval of the recommendations, a first draft of this guideline was written in accordance with the RIGHT and submitted to the Expert Guidance Group for approval. The recommendations of this guideline are planned to be updated within 3 years.

### 2.7 Supervision

This guideline was developed under the supervision of two methodologists.

### 2.8 Dissemination, implementation and evaluation

**Figure 2:**
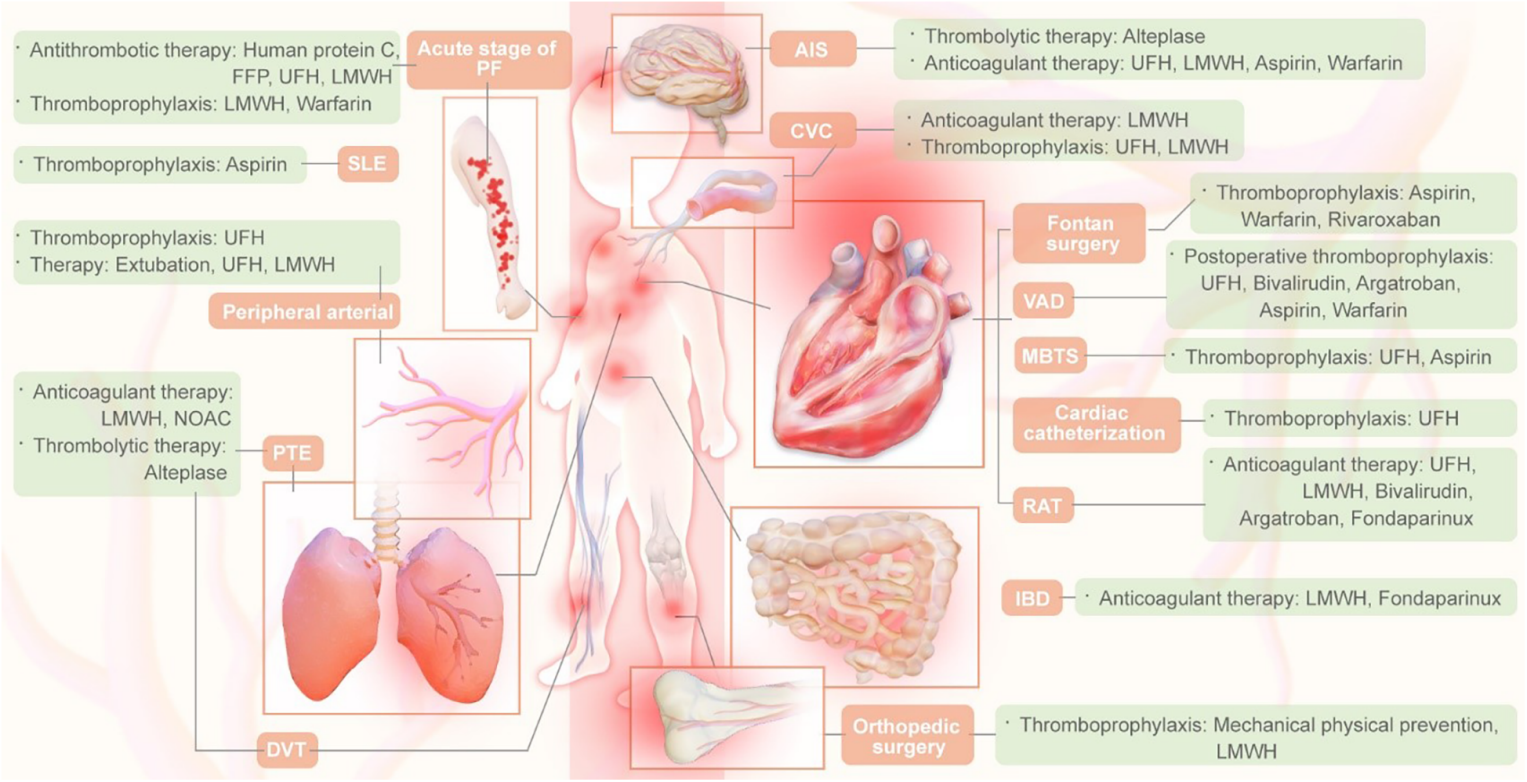
Thrombotic drug selection in children.

After the guideline is published, the Guideline Writing Group will disseminate and promote the guideline by participating in academic conferences, organizing special sessions for the guideline promotion, and evaluating the effectiveness of guideline implementation. In addition, the selection of antithrombotic drugs for children is detailed in Figure 2.

## 3 Specific recommendations for the pharmacological prevention and treatment of pediatric thrombosis

### 3.1 Recommendations for the prevention and treatment of pediatric VTE

Recommendation 1: (1) Anticoagulation is recommended for hemodynamically stable children with DVT and PTE. Systemic or cannulated thrombolytic therapy should be used only if organ or limb damage occurs due to large-vessel occlusion, contraindications to thrombolysis are ruled out, or hemodynamic instability occurs. rt-PA has become the preferred choice of thrombolytic therapy in pediatrics, and the representative drug is alteplase (strong recommendation, low quality evidence). (2) Anticoagulants are recommended for children with symptomatic DVT or PTE. Initial treatment (initial 5–10 days): for the initial acute phase treatment of children with newly diagnosed VTE, it is recommended to give at least 5 d of parenteral gastrointestinal anticoagulation using LMWH rather than UFH. subsequent treatment (after the initial 5–10 days): for children ≥12 years of age, it is recommended to use DOAC, e.g., rivaroxaban, dabigatran, for maintenance; for patients 2–12 years of age, the well-established evidence-based DOAC or LMWH; LMWH is recommended over other anticoagulants for patients <2 years of age (strong recommendation, moderate quality evidence).

Pediatric VTE is the most prevalent thrombotic disease in children, with deep venous thrombosis (DVT) being the most common type. Pulmonary thromboembolism (PTE) accounts for 5%–10% of pediatric thrombosis cases. The risk of VTE is highest in the neonatal period, then decreases, rising again around age 13 and reaching adult levels by age 16. Two peak incidences of pediatric VTE have been identified: the neonatal period (20%) and ages 11–18 (50%) (21, 22).

The optimal dosage of recombinant human tissue plasminogen activator (rt-PA) for systemic thrombolytic therapy of VTE in children remains unclear. Existing evidence is limited to case reports and single-center case series. Therefore, the guideline does not recommend routine thrombolytic therapy for pediatric VTE unless major vessel occlusion causes severe organ or limb damage or hemodynamic instability. If thrombolysis is necessary, t-PA or rt-PA should be used rather than other thrombolytic agents (23, 24). For catheter-directed thrombolysis with rt-PA, the recommended dosage is 0.015–0.2 mg·kg^−1^·h^−1^, with a median treatment duration of 24 h (25). Goldenberg et al. (26) found that low-dose [0.03–0.06 mg·kg^−1^·h^−1^] rt-PA infusion may reduce the incidence of major bleeding, with a maximum infusion duration of 96 h. Absolute contraindications for thrombolysis in children include active internal bleeding such as cerebrovascular infarction or hemorrhage, recent intracranial or spinal surgery or trauma, presence of intracranial aneurysms, vascular malformations or tumors, pericarditis, infective endocarditis, and a history of allergic reaction to contrast agents (27). When using rt-PA or t-PA, close monitoring of bleeding in children is required. Clinical laboratory indicators to be monitored include platelet count, fibrinogen levels, D-dimer, activated partial thromboplastin time (APTT), and prothrombin time (PT) (28–31).

It is recommended to use anticoagulants for children with symptomatic DVT or PTE. For asymptomatic DVT or PTE, the evidence for the use of anticoagulants is currently insufficient. The initial anticoagulant therapy of choice is LMWH which, compared to unfractionated heparin (UFH), has higher bioavailability, a longer half-life, and a more predictable anticoagulant effect. Generally, LMWH does not require laboratory monitoring in clinical use, but for special patient groups such as neonates and children, monitoring anti-Xa levels should be considered to adjust the dosage (Table 2). A systematic review/meta-analysis included 49 prospective and retrospective studies, encompassing 3,101 pediatric patients. Among children treated with therapeutic doses of LMWH, 79.9% achieved the therapeutic target with or without dose adjustment, the thrombus resolution rate was 63.5%, and the incidence of major bleeding complications was 1.8%. Among children receiving prophylactic LMWH, 90.4% achieved the prophylactic target, and the incidence of major bleeding was 0.6%. This suggests that LMWH is safe and effective for the treatment and prevention of VTE in children (34).

**Table 2:** Adjustment of LMWH dose according to anti-Xa levels in pediatric patients (32, 33).

Anti-Xa levels/ (U·ml^−1^)	Whether to delay next injection	Dose change	Repeated testing of anti-Xa levels
<0.35	No	Increase by 25%	4 h after next dose
0.35–0.49	No	increase by 10%	4 h after next dose
0.5–1.0	No	No change	Next day, then 1 week later and monthly thereafter while receiving reviparin-Na treatment (at 4 h after AM dose)
1.1–1.5	No	Decrease by 20%	Before next dose
1.6–2.0	After 3 h	Decrease by 30%	Before next dose then 4 h after next dose
>2.0	Discontinue until anti-Xa drops to 0.5 U·ml^−1^	Decrease by 40%	Before next dose, if not <0.5 U·ml^−1^ repeat q12 h

DOACs include direct thrombin inhibitors (dabigatran) and factor Xa inhibitors (rivaroxaban, apixaban, and edoxaban). Among them, dabigatran and rivaroxaban have been most well studied in children and have been approved for the treatment and secondary prevention of VTE in children in the United States, Canada, Europe, and other countries (35, 36). Rivaroxaban has been approved in China for the treatment and prevention of VTE in children. The EINSTEIN-Jr trial (37) was an open-label trial in which 500 children with VTE were randomized in a 2:1 ratio to rivaroxaban (weight-adjusted 20 mg equivalent after 5–9 days of parenteral therapy) or standard anticoagulation [continued use of UFH, LMWH, or switching to vitamin K antagonists (VKA)]. The majority of children in this trial were treated for 3 months, and children under 2 years of age received treatment for only 1 month. At the end of treatment, there was no recurrence of VTE in either group; venous recanalization was higher in the rivaroxaban group than in the control group (55% vs. 37%); there were 3 non-major bleeding events in the rivaroxaban group and no major bleeding events in either group. The DIVERSITY trial (38) was a randomized, controlled, open-label trial in 65 centers in 26 countries in which 267 children with VTE were randomized in a 2:1 ratio to dabigatran or standard anticoagulation [54% with VKA, 44% with LMWH, and 1% with fondaparinux]. After a median of 85 days of treatment, the primary composite efficacy endpoint (complete thrombus resolution rate, proportion of children without recurrent VTE, and VTE-related death) was similar in the dabigatran group to that in the control group (46% vs. 42%). The incidence of bleeding events was similar in the dabigatran and standard anticoagulant groups (22% vs. 24%), and the incidence of major bleeding events was the same (both 2%).

For most infants and young children (<2 years of age), the guideline recommends the use of LMWH. LMWH is superior to DOAC because LMWH has more experience with patients of this age, and its efficacy and safety are well established. In contrast, the efficacy and safety of DOACs in such patients remain uncertain because patients under 2 years of age are underrepresented in the pediatric DOAC trial. In the DIVERSITY trial (38), 267 children used dabigatran only in 22 patients ≤2 years of age, and the United States Food and Drug Administration (FDA) approved it only for minors aged 8–18 years. In children, LMWH is generally superior to VKA because dietary vitamin K intake in children varies considerably, VKA efficacy is more difficult to predict, and warfarin has only tablets, which makes it particularly difficult to use in infants and young children.

### 3.2 Recommendation on the prevention and treatment of CVC-related venous thrombosis in children

Recommendation 2: (1) For CVC catheter-related DVT, immediate extubation or removal of a central venous access device associated with thrombosis 3–5 days after therapeutic anticoagulation should be considered if the catheter is no longer functional, and LMWH anticoagulation may be preferred (weak recommendation, low quality evidence). (2) For HD children through AVF or CVC, it is recommended that UFH or LMWH be used for thromboprophylaxis. Citrate, rt-PA, or UFH may be used as the sealing solution at the end of dialysis (weak recommendation, low quality evidence).

Neonates develop VTE, 90% of which is induced by CVC (39). The incidence of CVC-related thrombi in neonates ranged from 1.1% to 66.7%, and the sites of formation were common in the liver, right atrium, and superior/inferior vena cava (40). The incidence of CVC-related thrombosis is 2%–81% in children and 20%–66% in children with CVC in China (41).

(1) Treatment of CVC-related VTE: the commonly used LMWH in children are enoxaparin and dalteparin, and the specific dosage can be adjusted according to the level of anti-factor Xa activity (42). Management of catheter-related DVT in hemodialysis (HD) patients is recommended to be the same as CVC-related DVT in non-HD patients. No study data are available regarding the safety and efficacy of NOAC in the treatment of VTE in children receiving HD. According to the ninth edition of the American College of Chest Physicians (ACCP) Guidelines for antithrombotic therapy in neonates and children (23), it is recommended that for children who have experienced DVT, if the catheter no longer works, it should be removed after 3–5 days of therapeutic anticoagulation in order to reduce the risk of embolism when the catheter is removed.
(2) Prevention of CVC-related VTE: pediatric CVC-related thrombotic anticoagulation studies are rare, and an open RCT has shown that prophylaxis with LMWH by subcutaneous injection twice daily is safe in most children (43). In children with HD, there is currently a lack of high-quality evidence for the prevention of catheter-related thrombosis, and a small study in children with HD using arteriovenous fistula (AVF) followed by subcutaneous injection of LMWH until AVF maturation (AVF is considered mature after successful placement of AVF and blood flow ≥5 ml·min^−1^) and compared with results without anticoagulation showed that the use of anticoagulant prophylaxis reduces the incidence of thrombosis (12.5% vs. 83%) (44). Anticoagulation of extracorporeal circuits should be determined by assessing the risk of bleeding vs. circuit thrombosis. The standard regimen includes a loading dose of 15–20 U·kg^−1^ UFH at the beginning of dialysis, followed by a continuous infusion of 10–20 U·kg^−1^·h^−1^, and stopping heparin infusion during the last 30 min of dialysis, with a target value of 1.5–2.0 times the normal range for APTT or an activated clotting time (ACT) of 180–220 s (45). LMWH can be used as an alternative treatment for UFH, and the ninth edition of the ACCP guidelines for antithrombotic therapy in neonates and children recommends the use of VKA or LMWH for thromboprophylaxis (23). If the patient has a coagulation disorder and is at high risk for bleeding, consider flushing the catheter with a 0.9% sodium chloride solution (46).

A recent study by Paglialonga et al. (47). evaluated the effect of warfarin on CVC preservation and related complications in children with end-stage renal disease (ESRD) undergoing chronic HD. It was found that prophylactic warfarin appears to be safe in children with chronic HD and may improve CVC retention in children at higher risk for CVC thrombosis. Argatroban, a direct thrombin inhibitor, was found to be more effective and safer in children with HD compared with UFH in two small randomized controlled studies (48, 49). Other anticoagulant options include fondaparinux and nafamostat. However, due to the lack of clinical evidence, current guidelines cannot make recommendations. Future studies are needed to assess and confirm the benefit and safety of primary thromboprophylaxis in children with HD.

Closure of catheters should be adequate after the end of dialysis, and most pediatric centers use heparin as a CVC sealer at concentrations that vary widely (1,000, 2,500, 5,000 U·ml^−1^) (50). In a small randomized controlled study, alteplase 1 mg·ml^−1^ in children was more effective in reducing intraluminal clot formation between HD courses compared with heparin 5,000 U·ml^−1^ (51).

### 3.3 Recommendations on prevention and treatment of peripheral arterial catheter thrombosis in children

Recommendation 4: (1) Continuous infusion of UFH 0.5 U·h^−1^ is recommended for prophylaxis of associated thrombosis in neonates and children using peripheral arterial catheters by dynamic assessment of the risk of high thrombosis (strong recommendation, high quality evidence). (2) For neonates and children with peripheral ductus arteriosus-related thrombosis, immediate catheter removal is recommended (weak recommendation, moderate quality evidence). (3) In neonates and children with symptomatic peripheral ductus arteriosus-related thrombosis, anticoagulant therapy with UFH (anti-factor Xa level 0.35–0.5 U·ml^−1^) or LMWH (anti-Xa level of 0.5–1.0 U·ml^−1^) is recommended (weak recommendation, low quality evidence).

Peripheral artery thrombosis in neonates and children is primarily associated with arterial puncture or catheterization, and leg growth retardation may occur with persistent partial or complete arterial occlusion (23). The current international consensus on the prevention and treatment of peripheral arterial catheter-related thrombosis in neonates and children is based on observational studies and/or clinical trials of limited quality and/or expert opinion with a very limited level of evidence.

Treatment of catheter-related arterial thrombosis in neonates and children included in a systematic review of 22 articles (52) mentions that UFH is the most commonly used anticoagulant (alone or in combination). A retrospective study (53) referred to a 52% incidence of peripheral catheter-related thrombosis and grouped and compared peripheral CAT regimens, i.e., 41 patients in the heparin alone (UFH/LMWH) plus aspirin group, 2 in the heparin (UFH/LMWH) plus VKA group, 2 patients in the aspirin alone group, and 1 in the VKA alone group. Heparin dose was adjusted by measuring anti-Xa levels (UFH anti-Xa levels of 0.35–0.5 U·ml^−1^ and 0.5–1.0 U·ml^−1^ for LMWH anti-Xa levels). The results showed that the switch from heparin to aspirin did not improve the cure rate of thrombosis in children, and heparins, particularly LMWH, are recommended as an effective anticoagulant option for the treatment of children without life-threatening ductus arteriosus-related thrombosis, and that children are advised to receive anticoagulant therapy for up to 4 weeks.

The ninth edition of the ACCP guidelines for antithrombotic therapy in neonates and children (23) recommends continuous infusion of UFH 0.5 U·h^−1^ for prevention of associated thrombosis for neonates and children using peripheral arterial catheterization. For neonates and children with peripheral ductus-related thrombosis, immediate catheter removal is recommended; for neonates and children with symptomatic peripheral ductus-related thrombosis, anticoagulation with UFH is recommended.

### 3.4 Recommendations on the prevention and treatment of thrombosis associated with fontan surgery in children

Recommendation 5: For children after Fontan surgery, the guideline recommends that aspirin, warfarin, or rivaroxaban prevent thrombosis (strong recommendation, moderate quality evidence).

Fontan surgery, or modified surgery, is a palliative surgical treatment for most congenital univentricular heart disease that transfers blood from the inferior and superior vena cava to the pulmonary artery (54). The incidence of postoperative thrombosis ranged from 3% to 16% (55), and thromboembolism was the major cause of early and late morbidity and death. Thromboembolism usually develops months to years after the Fontan surgery, with no identified predisposing factors, and despite aggressive treatment, thromboembolic mortality after Fontan surgery is high, so prevention of thromboembolism is important.

In 2011, a multicenter randomized controlled clinical trial (56) analyzed primary prophylactic antithrombotic regimens after Fontan surgery in children. 111 children were randomly assigned to aspirin (57, 5 mg·kg^−1^·d^−1^, no heparin) or heparin/warfarin (54, initially 24 h heparin followed by warfarin, target INR: 2.0–3.0) and antithrombotic prophylaxis for 2 years. In terms of efficacy, the overall thrombotic incidence was as high as 19% 2 years after Fontan surgery, and the cumulative risk of thrombosis persisted without significant difference between the two groups (2-year thrombotic event rate 24% in the heparin/warfarin group and 14% in the aspirin group, HR = 1.35, 95% CI: 0.62–3.00, *P* = 0.45); in terms of safety, there were a total of 2 deaths unrelated to thrombus and 1 major hemorrhage in each group. The minor bleeding rate was significantly higher in the heparin/warfarin group than in the aspirin group (*P* = 0.03), and there was no significant difference in other bleeding events. A 2013 study (57) conducted a secondary analysis of the above-mentioned RCT data to identify risk factors for thrombosis in the above-mentioned children, and the results showed that the incidence of venous thrombosis in children was as high as 69% in the 2.5 years after inclusion in the study. The results of multiple regression analysis showed that patients who consistently reached the INR target range (INR target range 2–3) or received aspirin had a lower risk of thrombosis than those who received aspirin (HR = 3.53, 95% CI: 1.35–9.20, *P* = 0.01), indicating that maintenance of INR compliance in warfarin-treated children is essential for thromboprophylaxis.

In 2013, a cohort study (58) analyzed 210 patients (median age 8.5 years at the time of surgery) undergoing the Fontan procedure at Boston Children's Hospital to assess and compare the effects of prophylactic aspirin and warfarin on thromboembolic events. Fifty percent of these patients did not receive thromboprophylaxis, and multiple regression analysis showed that no treatment with warfarin or aspirin was significantly associated with the occurrence of thromboembolic events (HR = 8.5, 95% CI: 3.6–19.9, *P* < 0.001) compared with warfarin or aspirin, whereas there was no significant difference between aspirin and warfarin (*P* = 0.768). In conclusion, prophylactic use of aspirin or warfarin was associated with a significant reduction in the incidence of thromboembolic events after the Fontan procedure, and the available evidence was not statistically different between the two treatments.

In 2019, a randomized, multicenter controlled trial (UNIVERSE trial) (59) assessed the safety and efficacy of rivaroxaban vs. aspirin for thromboprophylaxis in children after Fontan surgery. The study was divided into two parts, Part A and Part B, in which 12 patients were enrolled in a single-arm study, and the pharmacokinetics, pharmacodynamics, safety, and tolerability of rivaroxaban were evaluated to validate the selected pediatric dose. Part B evaluated the safety and efficacy of rivaroxaban vs. aspirin for thromboprophylaxis in children after Fontan surgery by including 100 children randomized 2:1 to rivaroxaban (suspension, individualized by body mass, comparable to the area under the curve for 24 h at 10 mg·d^−1^ in adults) and aspirin (5 mg·kg^−1^, qd) over a 12-month study period. The results of Part B showed that, in the rivaroxaban group, 1 (2%) child taking rivaroxaban experienced major bleeding (epistaxis requiring blood transfusion), 4 (6%) experienced clinically relevant non-major bleeding, and 21 (33%) had minor bleeding; in the aspirin group, 3 (9%) had clinically relevant non-major bleeding, and 12 (35%) had minor bleeding. In terms of efficacy, PTE occurred in 1 (2%) child in the rivaroxaban group; ischemic stroke occurred in 1 (3%) child in the aspirin group; and venous thrombosis occurred in 2 (6%) children. In conclusion, rivaroxaban was used for thromboprophylaxis after Fontan surgery compared to aspirin, with similar safety as aspirin, and efficacy aspects such as thrombotic events appeared to be less than aspirin but not statistically different (*P* = 0.095) (60). Currently, the FDA has approved rivaroxaban for thromboprophylaxis after Fontan surgery in children ≥2 years of age with congenital heart disease (35, 61).

In 2020, a multicenter cross-sectional study (62) analyzed the effect of aspirin and warfarin on the long-term prognosis of pediatric patients after Fontan surgery, and the results showed that cerebrovascular injury, including stroke (39%), and microbleeding (96%) were common complications after Fontan surgery. Warfarin administration was often associated with decreased bone health and increased bleeding. There was no significant difference in quality of life scores between warfarin and aspirin [warfarin (63.5 ± 18.5), aspirin (65.8 ± 16.7), *P* = 0.564]. Therefore, aspirin may be preferable as the primary long-term thromboprophylaxis agent, taking into account the compliance rate of INR and adverse events compared to aspirin.

### 3.5 Recommendations on prevention and treatment of thrombosis in ventricular assist devices (VAD) in children

Recommendation 6: Bivalirudine, or argatroban, is an alternative anticoagulant regimen for short-term postoperative thromboprophylaxis in children with contraindicated UFH who are advised to initiate UFH 8–48 h after the insertion of a VAD. Once hemodynamically stable has been assessed clinically, VKA can be bridged from parenteral anticoagulants (INR target range 2–3) or continued without bridging until cardiac transplantation or removal of VAD. Antiplatelet agents for thromboprophylaxis after VAD are an alternative, and aspirin can be initiated within 72 h of VAD insertion (strong recommendation, low quality evidence).

A VAD, as a bridge for transplantation or cardiac recovery, is commonly used in children with congenital or acquired heart failure-related diseases. The formation of VAD thrombi can lead to problems such as impaired device function, increased surgical complications, and increased cardiac instability. The probability of thromboembolic events in children with VAD is 9%–30% (63).

There are no high-quality clinical studies to assess the safety and efficacy of anticoagulant or antiplatelet regimens in children with VAD, and there is no standardized antithrombotic regimen. However, considering the catastrophic consequences of VAD circuit occlusion or embolic complications, anticoagulant and/or antiplatelet agents appear preferable to no medication. A 2018 literature review (64) summarized evidence related to antithrombotic therapy in pediatric patients with VAD and included a total of 27 studies involving a total of 558 children with VAD, mainly Berlin Heart EXCOR (87%) and Thoratec (7.5%). The results showed that the short-term anticoagulant drugs were mainly UFH, and the time to start anticoagulant therapy varied greatly, among which 10 studies started anticoagulation ≥24 h after implantation; aspirin in combination with dipyridamole was the most common antiplatelet regimen; and long-term anticoagulation regimens were UFH, LMWH, or warfarin.

Over time, antithrombotic regimens for children with EXCOR VAD have changed in favor of more aggressive antithrombotic therapy. A 2018 multicenter survey (65) analyzing the current status of antithrombotic practices in children receiving EXCOR VAD in Europe showed that 9 of the 18 hospitals surveyed reported early initiation of UFH within 6–20 h after surgery; aspirin was used as the first antiplatelet drug in 11 hospitals and clopidogrel as the second antiplatelet drug in 9 hospitals; the INR target range for warfarin use ranged from 2.5–3.5 in 10 hospitals; and a higher INR target range was used in 5 hospitals with an INR upper limit of 4.5.

In a multicenter retrospective study (66) in 2020, an analysis of 43 children (aged <19 years) treated with direct thrombin inhibitors (bivalirudine or argatroban) supported by *in vitro* VAD, including pulsatile and continuous flow VAD, showed overall transplant survival, major bleeding, and stroke rates of 88%, 16%, and 12%, respectively. This is the largest multicenter retrospective study of the use of direct thrombin inhibitor anticoagulation in pediatric VAD support, with a lower incidence of major bleeding and stroke events compared with other anticoagulants reported in pediatric VAD support in the literature. A single-center retrospective cohort study (67) in 2023 analyzed the outcome of anticoagulation with heparin and bivalirudin in children supported by the Berlin Heart EXCOR VAD and showed that bivalirudin exhibited higher anticoagulant stability compared with UFH; therapeutic anticoagulant effect was achieved earlier in the bivalirudin group (APTT) than in the UFH group (anti-Xa) (median, 5.7 and 69.5 h, respectively, *P* < 0.001); time in the treatment range was higher in the bivalirudin group than in the UFH group [52% (APTT) vs. 24% (APTT), *P* < 0.001; 52% (APTT) vs. 38% (anti-Xa), *P* = 0.003]. Although there were some differences in secondary outcomes, the rates of major bleeding, pump replacement secondary to significant thrombotic load, and stroke were similar in both groups.

### 3.6 Recommendations on prevention and treatment of thrombosis in children with modified blalock-taussig shunt (MBTS)

Recommendation 7: (1) UFH prophylaxis can be used in neonates and children undergoing MBTS (strong recommendation, low quality evidence). (2) Neonates and children after MBTS can be treated with aspirin 3–5 mg·kg^−1^·d^−1^ antithrombotic prophylaxis (strong recommendation, moderate quality evidence).

Single ventricle is a congenital heart defect that occurs due to fetal cardiac dysplasia during the first 8 weeks of pregnancy. During the first few years after birth, children may require a series of surgeries that alter the blood flow to increase the oxygen supply needed. The procedures included the following: MBTS, Glenn procedure, and Fontan procedure. The first procedure was a Blalock-Taussig shunt (B-T shunt), a palliative procedure used to enhance pulmonary blood flow (68). The incidence of Pediatric thrombosis with MBTS ranges from 0% to 40%, and overall mortality varies from 4.5% to 31.3% (69).

A multicenter, double-blind, randomized trial (CLARINET trial) conducted by Wessel et al. (70) in 2013 included 906 infants undergoing systemic pulmonary shunts (including MBTS, right ventricular-to-pulmonary shunts, central shunts, or arterial catheter stents, etc.) in combination with 0.2 mg·kg^−1^ clopidogrel on top of conventional therapy (with or without aspirin), with the primary composite endpoint of death occurring within 120 days of age, heart transplantation, shunt thrombosis, and cardiac surgery for events considered thrombotic nature. Finally, 467 patients were included in the test group and 439 in the placebo group, and 88% of the patients were prompted to use aspirin concomitantly after unblinding in both groups. The primary composite endpoint was not significantly different between the two groups (19.1% vs. 20.5%), clopidogrel treatment did not significantly benefit any subgroup, and the incidence of bleeding was similar between the two groups. Conclusions suggested that most infants after somatopulmonary shunt are treated with aspirin, and the combination of clopidogrel does not reduce any cause of mortality or shunt-related morbidity. A systematic review published in 2016 by Agarwal et al. (69) included 1,499 children from 15 cohort studies, most of whom were patients with MBTS. This systematic review concluded that although postoperative thromboprophylaxis appears to be the best strategy, whether long-term aspirin use is most effective remains controversial. The present findings lack the gold standard for thromboprophylaxis strategies, and more consistent conclusions are needed in the future.

Retrospective studies (71–73) suggest that standard weight-based aspirin administration may result in insufficient postoperative platelet inhibition in children. To assess whether the use of high-dose aspirin reduces shunt-related adverse events after MBTS, a single-center retrospective cohort study by Shah et al. included 34 infants under 1 year of age who received MBTS and aspirin in an intensive care unit (ICU). The investigator defined the aspirin treatment group as the standard dose (≥7 mg·kg^−1^, qd) and the high dose based on the initial dose (≥8 mg·kg^−1^, qd). The results showed that there was no significant difference in shunt intervention, shunt thrombosis or mortality between the two groups. In multiple logistic regression analysis, single ventricular morphology (OR = 5.2, 95% CI: 1.2–23, *P* = 0.03) and postoperative red blood cell transfusion ≥24 h (OR = 15, CI: 3–71, *P* < 0.01) were associated with shunt-related adverse events. It is suggested that high-dose aspirin may not be sufficient to reduce shunt-related adverse events in infants after MBTS, and future real-world studies are needed to determine the appropriate dose of aspirin in infants after MBTS (74).

The ninth edition of the ACCP guidelines for antithrombotic therapy in neonates and children (23) recommends intraoperative UFH prophylaxis in neonates and children with MBTS. For neonates and children after MBTS, aspirin or no antithrombotic prophylaxis is recommended compared with long-term use of LMWH or VKA. The Chinese expert consensus on surgical treatment of congenital heart disease: Serial surgeries for univentricular physiological corrections (75) proposed that in order to prevent thrombosis after B-T shunt surgery, it is recommended to take 3–5 mg·(kg·d)^−1^ aspirin postoperatively.

### 3.7 Recommendations on prevention and treatment of thrombosis in children undergoing cardiac catheterization

Recommendation 8: To prevent blood clots during heart catheterization in children, it is recommended to use heparin at a dose of 50–100 U/kg (max 5,000 U) and monitor ACT levels to keep them above 200 s. Infants under 12 months are at higher risk of clotting and should be closely monitored (strong recommendation, low quality evidence).

Cardiac catheterization has made significant progress in pediatrics over the past two decades, from a primary diagnostic tool to a primary treatment modality for children with congenital heart disease. Vascular complications, especially arterial thrombosis, are one of the most common adverse events after cardiac catheterization. The incidence of thrombosis in children undergoing cardiac catheterization ranged from 0.1% to 0.2% (76, 77). Anticoagulation with UFH should be routinely used after cardiac catheterization in children, unless there are specific contraindications (78). A review of the pathophysiology, prevention, and treatment of ATE formation after catheterization in children states that children with femoral artery thrombosis after cardiac catheterization are initially treated with UFH (79). When UFH alone is used, ATE is effective in 40%–70% of cases, depending on the duration of anticoagulation. Thrombolysis remains an appropriate strategy for children with persistent ischemia of the lower extremities after left heart catheterization, particularly those with femoral artery thrombosis that threatens extremities or organs and who do not respond to initial treatment for UFH.

Bulbul et al. (80) conducted a single-center study to assess whether higher doses of heparin would reduce the incidence of arterial thrombosis in children weighing ≤10 kg after cardiac catheterization. Sixty children were given 100 U·kg^−1^ heparin (group A) or 150 U·kg^−1^ heparin (group B) in A double-blind randomized fashion. After 20 min of heparin administration, the mean ACT in group A was significantly lower than that in group B (199 s vs. 251 s). Only 3 of 60 patients (5%, all in group B) required treatment for arterial thrombi. The study showed that administration of heparin at doses of 100 U·kg^−1^ or higher and ACT up to 200 s was relatively safe and did not need to reduce the incidence of arterial thrombosis in children after cardiac catheterization by increasing the heparin dose.

In 2011, a single-center, double-blind RCT (81, 82) objectively assessed the incidence of thrombotic events and bleeding during cardiac catheterization in both groups of children by clinical assessment and ultrasound. A total of 227 children were enrolled in the study, with an overall incidence of 4.6% thromboembolism and 6.6% bleeding, and arterial thrombosis occurring only in infants <12 months. High-dose (100 U·kg^−1^) and low-dose UFH (50 U·kg^−1^) were associated with thromboembolism (5% vs. 3%, RR = 1.5, 95% CI: 0.3–9) and bleeding (7% vs. 12%, RR = 0.6, 95% CI: 0.2–2). The study concluded that the incidence of thromboembolism and bleeding during cardiac catheterization is low in children, and the risk of arterial thrombosis is particularly high in infants. Low-dose UFH is sufficient for thromboprophylaxis during cardiac catheterization, so high-dose UFH is not superior to low-dose UFH in preventing thrombosis associated with cardiac catheterization.

In 2015, a cohort RCT (HEARTCAT study) (83) compared the efficacy of high-dose (100 U·kg^−1^) vs. low-dose UFH (50 U·kg^−1^) during cardiac catheterization in children. Blood samples were collected at 30, 60, and 90 min before and after administration to monitor the optimal parameters of UFH efficacy in children. The relationship between the dose of UFH and its anticoagulant effect, other factors affecting the effect of UFH, and the relationship between the efficacy of UFH and clinical prognosis were assessed by anti-Xa, APTT and ACT. The study included 242 samples from 149 children. Finally, the study showed that anti-Xa, APTT, and ACT showed good differences between different doses of UFH, and regression models showed that UFH efficacy was determined by UFH dose, age, baseline anti-Xa, baseline APTT, and baseline ACT level. Infants less than 12 months of age had a lower UFH effect than older children, and lower doses of UFH were more pronounced than higher doses of UFH, according to a study confirming a dose-dependent effect of age on the UFH effect.

In 2020, a Canadian retrospective case-control study (84) evaluated the use of low-dose heparin (<100 U·kg^−1^) vs. high-dose heparin (≥100 U·kg^−1^) given intravenously at the start of cardiac catheterization (i.e., immediate arterial puncture) with or without a maintenance dose of heparin to prevent postoperative arterial thrombosis in children. A total of 492 participants were included in two studies. This study showed no significant difference in the incidence of arterial thrombosis between the high and low dose heparin groups (RR low dose vs. high dose 1.06, 95% CI: 0.58–1.92; RD low vs. high dose 0.005, 95% CI: −0.04 to 0.05); There was no significant difference in the incidence of major bleeding events between the high-dose and low-dose UFH groups (low-dose vs. high-dose RR = 2.96, 95% CI for major bleeding events: 0.12–71.34). There was no significant difference in the incidence of DVT between the high and low dose heparin groups (low dose vs. high dose RR = 0.34, 95% CI: 0.01–8.28). Due to the limitations of current evidence, the small number of studies included in this study, and the lack of details reported in one study, it was not possible to determine the effect of different dosing regimens of UFH on vascular thrombosis during cardiac catheterization in children, and further randomized clinical trials are needed.

In conclusion, high-dose heparin is not superior to low-dose heparin in preventing catheter-related thrombosis in children. UFH requires ACT > 200 s, and infants younger than 12 months have a higher risk of arterial thrombosis after cardiac catheterization. However, there is still a lack of high-quality evidence of pharmacological antithrombotic regimens for the prevention and treatment of thrombosis after cardiac catheterization in neonates and children, and further studies are needed.

Current guidelines: the ninth edition of the ACCP guidelines for antithrombotic therapy in neonates and children (23), which recommends intravenous UFH for thromboprophylaxis rather than no prophylaxis or aspirin in neonates and children undergoing arterial cardiac catheterization, and recommends the use of 100 U·kg^−1^ heparin instead of 50 U·kg^−1^ heparin. For extended surgery, an increased heparin dose is recommended. 2013 American Heart Association Scientific Statement on the Prevention and Treatment of Thrombosis in Children and Congenital Heart Diseases (85): for thromboprophylaxis in neonates and children undergoing arterial cardiac catheterization, intravenous injection of 100 U·kg^−1^ UFH (maximum single dose not exceeding 5,000 U) is recommended to initiate programmed anticoagulation; programmed thromboprophylaxis with aspirin alone is not recommended; anticoagulation monitoring during cardiac catheterization in children recommends monitoring ACT after 1 h of dosing and every 30 min for longer procedures. If ACT does not meet the criteria, 50–100 U·kg^−1^ heparin should be given to maintain ACT > 200 s. The Chinese Guidelines for the Selection and Pharmaceutical Care of Anticoagulants for the Prevention and Treatment of VTE in 2021 (86) recommended that UFH or aspirin prophylaxis should be given to children undergoing cardiac catheterization.

### 3.8 Recommendations on prevention and treatment of perioperative thrombosis in pediatric orthopaedics

Recommendation 10: The incidence of VTE after hip surgery, pelvic and femoral osteotomies, elective spinal surgery, and posttraumatic VTE is low in children. Mechanical physical prophylaxis is recommended as well as LWMH for pharmacoprophylaxis (strong recommendation, low quality evidence).

Perioperative thrombosis in pediatric orthopaedic surgery can increase the risk of surgery, hinder blood flow, cause ischemia in the surgical area, cause tissue damage, and affect the postoperative rehabilitation process. Children after orthopaedic surgery may increase the risk of DVT and delay the recovery process due to prolonged bed rest and surgical site wound pain. Thrombi may also increase the risk of postoperative infection.

Georgopoulos et al. (87) found that the incidence of VTE after orthopedic surgery in children was 0.015%; Murphy found that the incidence of VTE after lower extremity or pelvic injury was 0.058% (88); Greenwald et al. (89) found that the incidence of DVT after pelvic or femoral fractures was 0.17% and that of DVT after spinal surgery in children was 0.0021%, but it should be noted that this risk increased 1.37-fold for each additional year of age. Currently, there are few specific guidelines on the prevention of VTE in children undergoing surgery (90), and most studies are based on expert opinion, single cases, or analyses from large DVT databases, with few specific data on pediatric orthopaedic surgery (91). A thrombus risk score available for trauma and pediatric orthopaedic surgery, incorporating 14 factors such as age (≥14 years of age or adolescence), history of DVT, immobilization for ≥3 days, and active cancer, was provided in a review, giving different scores. If the total score is ≤3, no other measures are required except for early mobilization and adequate water intake; if the score is ≥4, drug-based thromboprophylaxis should be initiated if there is no uncontrolled active bleeding (91).

The Association of Paediatric Anaesthetists of Great Britain and Ireland (APAGBI) Working Group on Guidelines for Childhood Thrombosis Prevention provided recommendations on perioperative care for Pediatric thrombosis (92). The incidence of hip surgery, pelvic and femoral osteotomies, elective spinal surgery, and posttraumatic VTE is low, and pharmacoprophylaxis of VTE in children is not recommended. Postpubertal adolescents (≥13 years of age) have a slightly increased risk of VTE and should be evaluated for prevention, and intervention may be required if other risk factors are present. In adolescents >13 years of age, thrombosis and bleeding risk should be assessed if limited mobility is expected to persist for >48 h. For those at low and intermediate risk of thrombosis, ensure water intake, early exercise, early removal of CVC, and consider mechanical prophylaxis, such as pharmacoprophylaxis recommended for high risk and no bleeding risk (93). For children with preexisting VTE, monotherapy with VKA, heparin, or rivaroxaban is recommended (86).

Mechanical methods include compression stockings, but no child-sized embolic stockings or intermittent pressure compression boots are available. Therefore, their use is limited to older and larger children, adolescents, and those with a body mass >40 kg. Must be worn during surgery until full recovery of mobility. The combination of mechanical and pharmacological prophylaxis reduces the overall risk of VTE compared to a single modality (93).

LMWH is the mainstay for the treatment and prevention of VTE in adults and children, where enoxaparin and dalteparin are available. In addition, anticoagulants have some effect on bone healing, and a large number of preclinical data suggest that many commonly used anticoagulants may have deleterious effects on bone healing, with heparin and warfarin being the first, followed by LMWH, and factor Xa inhibitors may pose the least risk, and some studies have even shown that they have the potential to enhance bone healing (94).

### 3.9 Recommendations on prevention and treatment of PF thrombosis in children

Recommendation 11: Testing the PROC or PROS1 gene is advised for neonatal PF to tailor drug therapy. For neonates with protein C deficiency, initial treatment may include human protein C concentrate or FFP with UFH or LMWH, followed by long-term anticoagulation with LMWH or warfarin (strong recommendation, low quality evidence).

PF is an acute, fatal disseminated intravascular coagulation (DIC) syndrome characterized by rapid progressive tissue hemorrhage and necrosis caused by cutaneous vascular thrombosis (11). PF is a comprehensive disease caused by a variety of predisposing factors and is associated with abnormal activation of procoagulant pathways, dysfunction of anticoagulant pathways, and endothelial cell injury, especially with the decrease of protein C and protein S (95). Neonatal PF is mainly caused by protein C or protein S deficiency caused by homozygous or compound heterozygous mutations in the PROC gene or PROS1 gene, with hereditary protein C deficiency being more common (23). Studies by Sirachainan et al. (96) show that the incidence of PF in neonates is about 2%. There are no high-quality clinical studies to provide a basis for the development of antithrombotic regimens for PF drugs in neonates. Therefore, the recommendations of this guideline are based on previous relevant clinical guidelines or case reports, and only references are provided for drug use recommendations.

During the acute phase of PF, the ninth edition of the ACCP guidelines for antithrombotic therapy in neonates and children (23) recommends that for neonates presenting clinically with homozygous protein C deficiency, it is suggested to administer protein C concentrate at a dose of 20–60 U·kg^−1^ until clinical manifestations resolve (typically lasting 6–8 weeks). If protein C concentrate is unavailable, fresh frozen plasma (FFP) is recommended at a dose of 10–20 ml·kg^−1^ q12 h. Goldenberg et al. (97), in combination with their clinical experience and previous literature reports, recommend the administration of protein C concentrate or FFP with or without heparin (to prevent the development of DIC) for antithrombotic treatment in neonatal PF patients during the acute phase (Table 3), but there is still a lack of high-quality clinical study support. There are no reports about protein S deficiency-induced PF in neonates.

**Table 3:** Recommendations for antithrombotic dose by goldenberg et al. (97).

Therapeutic agents	Dose	Monitoring objectives
Protein C concentrate	Loading dose 100 U·kg^−1^ Subsequently 50 U·kg^−1^ every 6 h	Protein C activity >50 U·dl^−1^ D-dimer normalized
FFP	10–15 ml·kg^−1^, q 8–12 h	Protein C activity >10 U·dl^−1^ D-dimer normalized
UFH	15–20 U·kg^−1^·h^−1^	Anti-Xa activity 0.3–0.7 U·ml^−1^
LMWH	1∼1.5 mg·kg^−1^·12 h^−1^	Anti-Xa activity 0.5–1.0 U·ml^−1^

Upon stabilization of the condition, the ninth edition of the ACCP guidelines for antithrombotic therapy in neonates and children (23) recommends that neonates with homozygous protein C deficiency may be treated long-term with VKA, LMWH, protein C replacement therapy, or liver transplantation. Given that protein C concentrates are not readily available, guidelines recommend long-term anticoagulation with LMWH or warfarin to prevent thrombosis. Anti-Xa activity should be monitored periodically when LMWH is used, and the anti-Xa activity should be controlled at 0.5–1.0 U·ml^−1^. INR values should be closely monitored when starting anticoagulation with warfarin until the INR reaches the quality target. The optimal therapeutic range is unknown, and the target range for INR is still recommended to be 2.0–3.0 (23). There have also been recent reports of significant efficacy of DOACs (long-term oral rivaroxaban 2.4 mg, q8 h in 3.5-year-old children, long-term edoxaban 1 mg·kg^−1^, qd in 4-year-old children, and long-term oral rivaroxaban 15 mg, qd in 13-year-old children) in place of warfarin to prevent thrombosis (98–100). DOACs can be used as an alternative therapy for warfarin-intolerant children, but there is still a lack of high-quality research support. Please refer to the actual situation of the child for administration and dosage.

### 3.10 Recommendations on prevention and treatment of IBD thrombosis in children

Recommendation 12: Prophylactic anticoagulant therapy is recommended for hospitalized children with severe IBD and a history of thrombosis, with LMWH as the preferred option. Therapeutic anticoagulant therapy should be given to children with IBD and thromboembolism, with LMWH as the first choice and fondaparinux as an alternative. (strong recommendation, low quality evidence).

IBD mainly includes two diseases: Crohn disease (CD) and ulcerative colitis (UC). Active IBD is associated with an increased risk of VTE, which occurs in 1%–2% of hospitalized children and occasionally includes PTE and cerebrovascular events (101–106). An observational study showed that 1% of hospitalized children and adolescents who had VTE, had major surgery (OR = 1.98, 95% CI: 1.54–2.55), or had a resultant hypercoagulability (OR = 7.39, 95% CI: 5.34–10.20) were at increased risk of developing VTE (106).

A retrospective study in 2013 (103), which included 532 children and young adults hospitalized for IBD, analyzed the characteristics of thromboembolism in children and young adults with IBD and showed an increased risk of thrombosis in pediatric inpatients hospitalized for IBD with colon involvement, and anticoagulant therapy in patients with active colitis of IBD appeared to be safe. While identifying hereditary thrombophilia and acquired risk factors in patients with IBD and thrombosis, the main risk factor appeared to be inflammatory status, and prophylactic anticoagulation was recommended for high-risk patients. Patients with IBD in this study were defined as a high-risk population for thrombosis if they had 1 primary criterion and 1 secondary criterion, which included admission for IBD with colon involvement or major surgery. Secondary criteria included personal history of thrombosis, first-degree family history of VTE, known thrombophilia, persistent antiphospholipid antibody (APL) >12 weeks, oral contraceptive use, smoking, obesity, thalidomide, or CVC.

Canadian Gastroenterology (107) and the European Organisation for Crohn's Disease and Colitis and the European Society for Childhood Gastroenterology, Liver Disease, and Nutrition (108) recommend the diagnosis of a first episode of VTE in children with clinically inactive IBD in the presence of irrelevant reversible predisposing factors and recommend anticoagulation for at least 3 months until risk factors have resolved for at least 1 month. For pediatric IBD patients diagnosed with first VTE in the presence of active disease, anticoagulant therapy is recommended until 3 months of IBD remission rather than stopping treatment at 3 months or indefinite anticoagulation. For pediatric IBD patients (younger than 18 years of age) without prior VTE, anticoagulant prophylaxis is advised not to be used if hospitalized for an IBD episode.

### 3.11 Recommendations on prevention and treatment of thrombosis in children with SLE

Recommendation 13: Aspirin should be considered for preventing thrombosis in children with SLE and high APL levels (weak recommendation, low quality evidence).

SLE is a chronic autoimmune disease characterized by the presence of autoantibodies and multiple organ involvement. Its clinical manifestations are highly heterogeneous. Childhood-onset SLE is specifically SLE that develops before the age of 18 years. At present, the global incidence rate is (0.3–2.5)/100,000 per year, the prevalence rate is (1.9–34.1)/100,000, and the male-female prevalence ratio is 1:4–5 (109). The prevalence of SLE in China is (30–70)/100,000. Childhood-onset SLE accounts for 10%–20% of the total number of SLE cases, and childhood-onset SLE accounts for 15%–25% of childhood rheumatism (110). There is a lack of high-quality evidence to guide the anticoagulant prevention and treatment of childhood SLE and antiphospholipid syndrome (APS), mainly referring to the European League against Rheumatism (EULAR) guidelines for the treatment of childhood SLE (111, 112).

A meta-analysis (113, 114) investigating the prophylactic effect of aspirin in APL-positive patients showed that patients taking aspirin had a significantly lower risk of developing a first thrombotic event. The 2017 European Initiative for the Sharing of Diagnosis and Treatment of Childhood-onset APS (112) recommends that in addition to hydroxychloroquine, the addition of antiplatelet drugs (e.g., aspirin) should be considered in the treatment of APL-positive childhood-onset SLE patients.

A retrospective study (115), which included 147 pediatric and adolescent patients (84% of whom were female) with a history of APS and thrombosis and analyzed the effect of anticoagulant therapy on thrombus recurrence in such patients, showed that patients with APS had a high risk of recurrent thrombosis and that children with primary APS were younger and had a higher incidence of arterial thrombotic events, whereas children with APS associated with underlying autoimmune diseases were older and had a higher frequency of venous thrombotic events associated with hematologic and cutaneous manifestations, so prophylactic anticoagulation was warranted. A multicenter study (116) used data on the clinical manifestations of APS in children to determine treatment impact and long-term outcomes, which showed a high incidence of thrombosis in APS, justifying consideration of anticoagulation in all pediatric patients with confirmed APS. Data from several additional cohort studies (117–119) show the potential benefit of antiplatelet agents in the primary prevention of thrombotic events in SLE patients with persistently positive APL with moderate to high titers.

In 2021, a single-center retrospective study in China (120) analyzed the clinical characteristics of pediatric patients with SLE complicated by cerebral venous sinus thrombosis, and the combination of SLE treatment with anticoagulant therapy (LMWH, warfarin, rivaroxaban, aspirin) could improve the prognosis of cerebral venous sinus thrombosis in pediatric SLE patients in detail. One of the five patients was cured, and the remaining four had a thrombus reduction.

The French National Programme for the Diagnosis and Care of APS in Adults and Children (121) mentions that first-line treatment for APS with thrombotic events includes heparin, long-term anticoagulant therapy, and VKA (usually warfarin). Long-term secondary prevention of VKA prevents recurrent thrombosis. The INR goals for secondary prevention of intravenous APS are 2–3, and those for arterial APS are 3–4 or 2–3 in combination with low-dose aspirin.

In addition, the recommendations in this guideline for ischemic stroke and cardiac thrombosis can be obtained from the Supplementary Material.

## 4 Discussion and summary

The compass presents 13 recommendations for the use of antithrombotic drugs in neonates and/or children with thrombosis on 13 clinical issues. Neonatal and pediatric thrombosis is an increasingly medically recognized disease, and most children do not develop spontaneous thrombosis compared to adults, so once risk factors are eliminated, the long-term risk of recurrence is small. Due to the peculiarity of the pediatric population, the current clinical medical evidence is still scarce. In pediatric use, most physicians will use off-label drugs according to guidelines, expert consensus, case reports, etc., but the risk of adverse drug reactions in children is significantly increased. Additionally, due to the extremely low quality of evidence retrieved regarding thrombosis associated with the placement of artificial heart valves and cardiomyopathy, the guideline does not provide specific recommendations. However, recommendations from other guidelines are available for reference (23, 122, 123). In addition, guidelines encourage the organization of multidisciplinary care and individualized use of drugs for children as necessary during the prevention and treatment of thrombosis in children. Comprehensive evaluation of children's disease and the development of an individualized treatment plan can reduce misdiagnosis and missed diagnoses, optimize therapeutic effects, improve prognosis, reduce complications in children, and promote knowledge sharing and communication at the same time.

The guideline serves as academic guidance recommendations only and not as a legal basis. The guideline is not mandatory and does not serve as the basis for the identification of medical malpractice and the identification of medical responsibility, and are only for the reference of relevant healthcare professionals involved in the prevention and treatment of children's thrombosis in medical institutions. In clinical practice, the specific clinical treatment scheme varies from person to person. With the development of medical science and technology, the contents of the guideline will be further improved.

## Group member of [Expert group on the development of the guideline]

**Expert Guidance Group (sorted by last name in pinyin):**

Basang Lhamo People's Hospital of the Tibet Autonomous Region, Li Chen West China Second Hospital of Sichuan University, Qi Chen Guizhou Provincial People's Hospital, Xiao Chen The First Affiliated Hospital of Sun Yat-sen University, Yalin Dong The First Affiliated Hospital of Xi'an Jiaotong University, Weiyi Feng The First Affiliated Hospital of Xi'an Jiaotong University, Weihong Ge Nanjing Drum Tower hospital, Daihong Guo Chinese PLA General Hospital, Ruichen Guo Qilu Hospital of Shandong University, Yujin Guo Jining First People's Hospital, Jinhan He West China Hospital of Sichuan University, Yilan Huang Affiliated Hospital of Southwest Medical University, Yuechuan Jia General Hospital of Ningxia Medical University, Ling Jiang The First Affiliated Hospital of University of Science and Technology of China, Jinqi Li Sichuan Academy of Medical Sciences & Sichuan Provincial People's Hospital, Shuhong Liang The First Affiliated Hospital of Zhengzhou University, Houwen Lin Renji Hospital affiliated to Shanghai Jiaotong University School of Medicine, Yang Lin Beijing Anzhen Hospital of Capital Medical University, Dong Liu Tongji Hospital, School of Medicine, Tongji University, Enwu Long Sichuan Academy of Medical Sciences & Sichuan Provincial People's Hospital, Manling Ma The First Affiliated Hospital of Harbin Medical University, Liyan Miao The First Affiliated Hospital of Soochow University, Xiaoyong Qi Hebei General Hospital, Yan Qian The Second Affiliated Hospital of Chongqing Medical University, Feng Qiu The First Affiliated Hospital of Chongqing Medical University, Qiang Su Nanchong Central Hospital, The Affiliated Nanchong Central Hospital of North Sichuan Medical College/Nanchong Hospital of Beijing Anzhen Hospital Capital Medical University, Fengbo Wu West China Hospital of Sichuan University, Xinan Wu The First Hospital of Lanzhou University, Yong Yang Sichuan Academy of Medical Sciences & Sichuan Provincial People's Hospital, BaotaoYu The General Hospital of Western Theater Command of PLA, Linan Zeng West China Second University Hospital, Sichuan University, Bikui Zhang The Second Xiangya Hospital of Central South University, Jian Zhang Xinhua Hospital Affiliated to Shanghai Jiao Tong University School of Medicine, Kanghuai Zhang The Second Affiliated Hospital of Xi'an Jiaotong University, Mao Zhang Sichuan Academy of Medical Sciences & Sichuan Provincial People's Hospital, Wei Zhang Henan Provincial People's Hospital, Rongsheng Zhao Peking University Third Hospital, Zhigang Zhao Beijing Tiantan Hospital of Capital Medical University, Yingli Zheng Fuwai Hospital, Chinese Academy of Medical Sciences.

**Expert Advisory Group (sorted by last name in pinyin):**

Jinlan Chen The Second Xiangya Hospital of Central South University, Xiaoling Cheng Beijing Children's Hospital Affiliated to Capital Medical University, Dasheng Dang General Hospital of Northern Theater Command of PLA, Xiangyu Ding Hebei Children's Hospital, Shujie Dong Peking University Third Hospital, Xiaoming Du Shengjing Hospital of China Medical University, Yu Gao Chengdu Women and Children's Hospital, Lu Guo Sichuan Academy of Medical Sciences & Sichuan Provincial People's Hospital, Yong Han Union Hospital, Tongji Medical College, Huazhong University of Science and Technology, Yang Hu Peking Union Medical College Hospital, Huang Kui Sichuan Academy of Medical Sciences & Sichuan Provincial People's Hospital, Liang Huang West China Second Hospital of Sichuan University, Yuntao Jia Children's Hospital Affiliated to Chongqing Medical University, Haiyan Lao Guangdong Provincial People's Hospital, Bo Li Hebei Children's Hospital, Boxia Li First Hospital of Lanzhou University, Gang Li Sichuan Academy of Medical Sciences & Sichuan Provincial People's Hospital, Gen Li Sichuan Provincial Maternity and Child Health Hospital, Zhiling Li Shanghai Children's Medical Center Affiliated to Shanghai Jiaotong University School of Medicine, Shengzhong Liu Sichuan Academy of Medical Sciences & Sichuan Provincial People's Hospital, Yan Mou The First Hospital Affiliated to Shandong Medical University, Minghui Pang Sichuan Academy of Medical Sciences & Sichuan Provincial People's Hospital, Bing Shu The First Affiliated Hospital of University of Science and Technology of China, Na Wang Second Affiliated Hospital of Chongqing Medical University, Yunyun Wei Beijing Children's Hospital Affiliated to Capital Medical University, Hang Xu Gulou Hospital Affiliated to Nanjing University School of Medicine, Ping Xu The second xiangya hospital of central south university, Ming Yang Affiliated Hospital of North Sichuan Medical College, Tao Yu Sichuan Academy of Medical Sciences & Sichuan Provincial People's Hospital, Jiaxing Zhang Guizhou Provincial People's Hospital, Jinhua Zhang Maternity and Child Health Hospital College of Clinical Medicine for Obstetrics & Gynecology and Pediatrics, Fujian Medical University, Li Zhang The Second Affiliated Hospital of Xi ‘an Jiaotong University, Lu Zhang The First Affiliated Hospital of Xi'an Jiaotong University, Zhe Zhang The Second Affiliated Hospital of Army Medical University, Ping Zheng Nanfang Hospital of Nanfang Medical University, Jintuo Zhou Fujian Maternity and Child Health Hospital College of Clinical Medicine for Obstetrics & Gynecology and Pediatrics, Fujian Medical University, Yang Zhou Beijing Anzhen Hospital Affiliated to Capital Medical University, Ying Zhou Peking University First Hospital, Zhengyi Zhu Children's Hospital Affiliated to Zhejiang University School of Medicine.
