# Supplementary material for: Addressing challenges in pediatric thrombosis: a comprehensive guideline development
Source: Front Pediatr. 2025 Jan 27;13:1519517. doi: 10.3389/fped.2025.1519517 (PMC11841464; doi:10.3389/fped.2025.1519517)
Supplement: Supplementary file 1 [file Datasheet1.docx]

**Supplementary materials**

**Supplement to Recommendations**

**12 Recommendations on prevention and treatment of ischemic stroke in children**

**Recommendation 3: (1) Thrombolytic therapy is recommended within 4.5 h of the onset of AIS symptoms, and alteplase is recommended for thrombolytic drugs (weak recommendation, very low quality evidence). (2) Children who are not eligible for thrombolytic therapy are initially given UFH, LMWH, or aspirin until head-carotid artery dissection or cardiogenic thrombosis are excluded, and then aspirin 1 ~ 5 mg·kg^-1^·d^-1^ maintenance treatment is continued. Anticoagulant therapy should be selected for children with confirmed head-carotid artery dissection or cardiogenic ischemic stroke with UFH, LMWH, and warfarin (weak recommendation, low quality evidence).**

Acute ischemic stroke (AIS) in children is a rare and complex disease. In developed countries, the annual incidence of childhood stroke is (3 ~ 25)/100,000. The incidence of AIS is highest in infants and children < 5 years of age(Mallick et al., 2014; Ferriero et al., 2019; Li et al., 2021). Its occurrence is usually associated with a variety of factors, such as arterial disease (e.g., intracranial arteriosclerosis or arteritis), cardiovascular disease (especially congenital heart disease), and hematologic disorders (such as sickle cell disease or coagulation abnormalities). Because of the multiple etiologies of ischemic stroke in children, a comprehensive medical assessment is required to identify the etiology and develop treatment plans(McKinney et al., 2018; Hongyu, 2022). Amlie-Lefond et al.(Amlie-Lefond et al., 2020) collected data from 16 children with ischemic stroke treated with intravenous rt-PA in a retrospective Pediatric Stroke Thrombolysis Trial in 2020, and the results suggest that children with AIS have a low overall risk of symptomatic intracranial hemorrhage after receiving intravenous rt-PA within 4.5 h of onset. Approximately 2% of children with AIS in the United States have been treated with rt-PA; currently, thrombolytic therapy is mainly used in children ≥ 2 years of age, and the safety and efficacy of intravenous rt-PA in children < 2 years of age are unknown(Bernard et al., 2014; Rivkin et al., 2016). In a review of pediatric AIS, Mastrangelo M et al.(Mastrangelo et al., 2022) noted that alteplase can be used in children who meet all four of the following criteria at a dose of 0.9 mg·kg^-1^ (maximum 90 mg), 10% bolus injection within the first 5 min, and the remaining 90% intravenously within 1 h: (1) within 4.5 h of known symptom onset; (2) > 2 years; (3) acute focal neurological deficits and a National Institutes of Health Child Stroke Scale score ≥ 6 points and ≤ 24 points; (4) no contraindications. If any of the above 4 criteria is not met, rt-PA cannot be used for thrombolysis, and aspirin is recommended for children. The specific regimen is: for the first 14 d, 5 mg·kg^-1^, and the maximum dose is 300 mg at 24 h; after 14 d, 1 mg·kg^-1^, up to 75 mg within 24 h.

At present, antithrombotic therapy is the main treatment in the acute phase and the secondary prevention in the chronic phase of AIS. Antithrombotic therapy includes antiplatelets (representing the drug aspirin) and anticoagulants (UFH, LMWH, and warfarin). A nonrandomized prospective study comparing the effects of LMWH and aspirin in the secondary prevention of AIS in children found that both showed advantages in preventing relapse. Retrospective studies have shown that significant bleeding events with anticoagulant therapy in pediatric AIS are rare, but the risk of transformation from acute ischemic intracerebral hemorrhage needs to be considered(Felling et al., 2017).

The American Heart Association recommends anticoagulants for extracranial dissection and cardiogenic stroke, whereas aspirin is recommended for idiopathic stroke(Roach et al., 2008). RCT studies of anticoagulants in children with AIS in the acute phase are lacking, and UFH, LMWH, or aspirin are recommended for the initial treatment of children with AIS before the diagnostic evaluation is completed. The ninth edition of the ACCP guidelines for antithrombotic therapy in neonates and children(Monagle et al., 2012) recommends initial anticoagulant therapy or aspirin 1 ~ 5 mg·kg^-1^·d^-1^ until head-carotid artery dissection or cardiogenic emboli are ruled out, followed by low-dose aspirin maintenance therapy for pediatric stroke associated with non-sickle cell disease. The Scientific Statement for the Management of Stroke in Infants and Children(Roach et al., 2008), the ACCP Eighth Edition Antithrombotic Therapy Guidelines for Neonates and Children(Monagle et al., 2008), and the Guidelines for Investigation and Management of Stroke and Neurovascular Diseases(Fuyong et al., 2012) recommend that anticoagulant therapy (LMWH or warfarin at 3 ~ 6 months) be considered for patients with moyamoya disease or moyamoya syndrome because of the risk of spontaneous intracerebral hemorrhage. Anti-Xa levels should be monitored in children with UFH ranging from 0.35 ~ 0.7 U·mL^-1^(Monagle et al., 2012).

**13 Recommendations on prevention and treatment of** **cardiac thrombosis in children**

**Recommendation 9: For RAT in children, UFH, LMWH, and oral anticoagulants may be selected on a case-by-case basis, and bivalirudine, argatroban, and fondaparinux sodium may be selected with caution in children with platelet dysfunction, platelet count, or heparin-induced thrombocytopenia (weak recommendation, very low quality evidence).**

Right Atrial Thrombosis (RAT), a common complication of indwelling CVC in infants and children, has been shown to be associated with CVC in 91% of pediatric RAT cases(Bagwell and Marchildon, 1989; Berman et al., 1991; Yang et al., 2010). RAT-related studies in infants and children are mainly from retrospective cases, mostly case reports. The treatment methods reported in the literature mainly include surgical thrombectomy, drug thrombolysis, anticoagulant therapy, or observation alone. Anticoagulant therapy is one of the most commonly used methods, and the main anticoagulants used are UFH, LMWH, warfarin, bivalirudine, argatroban, rivaroxaban, and dabigatran(T et al.; Wacker et al., 1994; Cesaro et al., 2002; Bronzetti et al., 2009; Yang et al., 2010; Shin et al., 2013; Tincher and Cox, 2013; Baykan et al., 2014; Cetin et al., 2014; Zharkov et al., 2017; Ma et al., 2018; Panagopoulos et al., 2018; Chunduri et al., 2019; Lulu et al., 2019; Nurmeev et al., 2019; Schapkaitz et al., 2019; Bosch and Albisetti, 2020; Yan and Li, 2020; Aghaei Moghadam et al., 2021; Jarvis et al., 2021; Doya et al., 2022). Outcome measures were thrombotic changes or adverse outcomes. There are no high-quality studies to assess the safety and efficacy of anticoagulant or antiplatelet therapy in pediatric RAT, and there is no standardized antithrombotic regimen.

A retrospective single-center cohort study(Bosch and Albisetti, 2020) conducted in 2020 compared the outcomes of anticoagulant therapy versus non-anticoagulant therapy in patients with RAT (aged 2 d to 20 years). In the anticoagulant group, 27 patients received treatment, with the most commonly used medications being enoxaparin, UFH, aspirin, bivalirudin, and warfarin. In the non-anticoagulant group, 29 patients did not receive treatment. The results indicated that in the treatment group, 14 patients developed complications associated with adverse outcomes, and 6 patients died. In contrast, in the non-treatment group, only 4 patients developed complications associated with adverse outcomes, and 2 patients died. Additionally, thrombus progression was more frequent in the anticoagulant group. For patients with low-risk RAT, it should be assessed whether anticoagulant therapy can be avoided.

A retrospective multicenter study in 2021(Jarvis et al., 2021) assessed the incidence, treatment, and outcome of asymptomatic right atrial thrombi detected by routine echocardiography in Nordic and Baltic countries after treatment of childhood acute lymphoblastic leukemia using the Nordic Society of Pediatric Hematology and Oncology Lymphocytic Leukemia 2008 protocol. Eleven of 406 patients had asymptomatic right atrial thrombi, 7 of whom had CVC removed prior to echocardiography, 3 patients with indwelling CVC treated with anticoagulant (LMWH), and 1 patient without anticoagulant, and as a result, none of the thromboses affected cardiac function, showed no signs of progression at follow-up, and showed no spontaneous or treatment-related regressions.

**References**

Aghaei Moghadam, E., Mahmoudi, S., Safari Sharari, A., Afsharipour, M., Gorji, M., Navaeian, A., et al. (2021). Giant intracardiac thrombosis in an infant with leukaemia and prolonged COVID-19 viral RNA shedding: a case report. *Thromb J* 19(1)**,** 31. doi: 10.1186/s12959-021-00285-8.

Amlie-Lefond, C., Shaw, D.W.W., Cooper, A., Wainwright, M.S., Kirton, A., Felling, R.J., et al. (2020). Risk of Intracranial Hemorrhage Following Intravenous tPA (Tissue-Type Plasminogen Activator) for Acute Stroke Is Low in Children. *Stroke* 51(2)**,** 542-548. doi: 10.1161/strokeaha.119.027225.

Bagwell, C.E., and Marchildon, M.B. (1989). Mural thrombi in children: potentially lethal complication of central venous hyperalimentation. *Crit Care Med* 17(3)**,** 295-296.

Baykan, A., Ozyurt, A., Korkmaz, L., Pamukcu, O., Argun, M., Ozturk, A., et al. (2014). Giant right atrial thrombus in premature newborn. *J Thromb Thrombolysis* 37(3)**,** 353-355. doi: 10.1007/s11239-013-0936-6.

Berman, W., Jr., Fripp, R.R., Yabek, S.M., Wernly, J., and Corlew, S. (1991). Great vein and right atrial thrombosis in critically ill infants and children with central venous lines. *Chest* 99(4)**,** 963-967. doi: 10.1378/chest.99.4.963.

Bernard, T.J., Rivkin, M.J., Scholz, K., deVeber, G., Kirton, A., Gill, J.C., et al. (2014). Emergence of the primary pediatric stroke center: impact of the thrombolysis in pediatric stroke trial. *Stroke* 45(7)**,** 2018-2023. doi: 10.1161/strokeaha.114.004919.

Bosch, A., and Albisetti, M. (2020). Management of Venous Thromboembolism in Children: Current Recommendations and Therapeutic Options. *Ther Clin Risk Manag* 16**,** 673-679. doi: 10.2147/tcrm.S218622.

Bronzetti, G., D'Angelo, C., Angelini, A., Picchio, F.M., and Boriani, G. (2009). Resolution of atrial thrombosis with heparin in a newborn with atrial flutter. *Acta Paediatr* 98(7)**,** 1211-1214. doi: 10.1111/j.1651-2227.2009.01235.x.

Cesaro, S., Paris, M., Corrò, R., Svaluto, G., Zanon, G.F., Gamba, P., et al. (2002). Successful treatment of a catheter-related right atrial thrombosis with recombinant tissue plasminogen activator and heparin. *Support Care Cancer* 10(3)**,** 253-255. doi: 10.1007/s00520-001-0328-x.

Cetin, I., Ekici, F., Ünal, S., Kocabaş, A., Sahin, S., Yazıcı, M.U., et al. (2014). Intracardiac thrombus in children: the fine equilibrium between the risk and the benefit. *Pediatr Hematol Oncol* 31(5)**,** 481-487. doi: 10.3109/08880018.2014.919546.

Chunduri, S., Behera, A., and Seshagiri, K. (2019). Right Atrial Thrombus in a Preterm Infant. *Indian Heart Journal* 71**,** S74. doi: 10.1016/j.ihj.2019.11.162.

Doya, L.J., Alyousef, K., Oukan, M., Razzok, A., Alshabab, B.S., AlEid, T., et al. (2022). Clear cell sarcoma of the kidney with inferior vena cava thrombus: a case report. *J Med Case Rep* 16(1)**,** 295. doi: 10.1186/s13256-022-03489-2.

Felling, R.J., Sun, L.R., Maxwell, E.C., Goldenberg, N., and Bernard, T. (2017). Pediatric arterial ischemic stroke: Epidemiology, risk factors, and management. *Blood Cells Mol Dis* 67**,** 23-33. doi: 10.1016/j.bcmd.2017.03.003.

Ferriero, D.M., Fullerton, H.J., Bernard, T.J., Billinghurst, L., Daniels, S.R., DeBaun, M.R., et al. (2019). Management of Stroke in Neonates and Children: A Scientific Statement From the American Heart Association/American Stroke Association. *Stroke* 50(3)**,** e51-e96. doi: 10.1161/str.0000000000000183.

Fuyong, J., Xiangyang, G., Li, W., and Jing, L. (2012). Advances in the diagnosis and treatment of stroke in children. *Journal of Practical Pediatrics* (12)**,** 940-943. doi: 10.3969/j.issn.1003-515X.2012.12.017.

Hongyu, W. (2022). Advances in the diagnosis and treatment of arterial ischemic stroke in children. *Hebei Pharmaceuticals* 44(16)**,** 2526-2531.

Jarvis, K.B., Andersson, N.G., Giertz, M., Järvelä, L., Lindinger, O., Långström, S., et al. (2021). Asymptomatic Right Atrial Thrombosis After Acute Lymphoblastic Leukemia Treatment. *J Pediatr Hematol Oncol* 43(4)**,** e564-e566. doi: 10.1097/mph.0000000000001848.

Li, J., Qinrong, H., and Nong, X. (2021). Advances in clinical diagnosis of stroke in children. *Healthcare Medicine Research and Practice* 18(06)**,** 176-180+188.

Lulu, R., Feng, C., Xiaolong, C., Xiaobin, L., Yewei, X., Rufang, Z., et al. (2019). A case of multisystem Langerhans cell hyperplasia in a child with combined multiple thrombosis and review of the literature. *Chinese Journal of Pediatric Hematology and Oncology* (05)**,** 254-259. doi: 10.3969/j.issn.1673-5323.2018.05.007.

Ma, J.Y., Zhang, X., Li, X.F., He, L.J., Ma, N., Wei, Y.Y., et al. (2018). Thrombotic storm in a 4-year-old boy with a thrombus in the right atrium. *Int J Immunopathol Pharmacol* 32**,** 2058738418778121. doi: 10.1177/2058738418778121.

Mallick, A.A., Ganesan, V., Kirkham, F.J., Fallon, P., Hedderly, T., McShane, T., et al. (2014). Childhood arterial ischaemic stroke incidence, presenting features, and risk factors: a prospective population-based study. *Lancet Neurol* 13(1)**,** 35-43. doi: 10.1016/s1474-4422(13)70290-4.

Mastrangelo, M., Giordo, L., Ricciardi, G., De Michele, M., Toni, D., and Leuzzi, V. (2022). Acute ischemic stroke in childhood: a comprehensive review. *Eur J Pediatr* 181(1)**,** 45-58. doi: 10.1007/s00431-021-04212-x.

McKinney, S.M., Magruder, J.T., and Abramo, T.J. (2018). An Update on Pediatric Stroke Protocol. *Pediatr Emerg Care* 34(11)**,** 810-815. doi: 10.1097/pec.0000000000001653.

Monagle, P., Chalmers, E., Chan, A., deVeber, G., Kirkham, F., Massicotte, P., et al. (2008). Antithrombotic therapy in neonates and children: American College of Chest Physicians Evidence-Based Clinical Practice Guidelines (8th Edition). *Chest* 133(6 Suppl)**,** 887s-968s. doi: 10.1378/chest.08-0762.

Monagle, P., Chan, A.K., Goldenberg, N.A., Ichord, R.N., Journeycake, J.M., Nowak-Göttl, U., et al. (2012). Antithrombotic therapy in neonates and children: antithrombotic therapy and prevention of thrombosis: American College of Chest Physicians Evidence-Based Clinical Practice Guidelines. *Chest* 141(2)**,** e737S-e801S.

Nurmeev, I.N., Kostromin, A.A., and Okoye, B. (2019). Treatment of the newborn with a floating thrombus in the right atrium: Case report. *Russian Open Medical Journal* 8(1). doi: 10.15275/rusomj.2019.0111.

Panagopoulos, D., Loukopoulou, S., Karanasios, E., Grigoriadou, G., and Eleftherakis, N. (2018). Cerebral hemorrhagic infarction as the initial manifestation of deep venous thrombosis in a child with patent foramen ovale. *Glob Cardiol Sci Pract* 2018(2)**,** 17. doi: 10.21542/gcsp.2018.17.

Rivkin, M.J., Bernard, T.J., Dowling, M.M., and Amlie-Lefond, C. (2016). Guidelines for Urgent Management of Stroke in Children. *Pediatr Neurol* 56**,** 8-17. doi: 10.1016/j.pediatrneurol.2016.01.016.

Roach, E.S., Golomb, M.R., Adams, R., Biller, J., Daniels, S., Deveber, G., et al. (2008). Management of stroke in infants and children: a scientific statement from a Special Writing Group of the American Heart Association Stroke Council and the Council on Cardiovascular Disease in the Young. *Stroke* 39(9)**,** 2644-2691. doi: 10.1161/strokeaha.108.189696.

Schapkaitz, E., Weil, R., White, D., and Klugman, S. (2019). Asymptomatic catheter-related venous thrombosis in a child with cystic fibrosis: When to treat? *J Vasc Nurs* 37(1)**,** 43-45. doi: 10.1016/j.jvn.2018.09.001.

Shin, J.H., Kim, G.N., Kil, H.R., and Chang, M.Y. (2013). Successful use of low molecular weight heparin in intracardiac thrombus of an extremely low birth weight infant. *Indian J Pediatr* 80(9)**,** 779-780. doi: 10.1007/s12098-012-0840-7.

T, B.B., William, R., Grace, F., Earnest Amankwah, Austin Sellers, David Procaccini, et al. *Outcomes of right atrial thrombi in children; an observational cohort study* [Online]. Available: <https://aspho.planion.com/Web.User/AbstractDet?ACCOUNT=ASPHO&ABSID=16240&CONF=AM20&ssoOverride=OFF&CKEY>= [Accessed 2023-02-24 2023].

Tincher, L., and Cox, K. (2013). Treatment of a pediatric patient with dabigatran. *Critical Care Medicine* 41(12)**,** A343-A344. doi: 10.1097/01.ccm.0000440561.67860.b5.

Wacker, P., Oberhansli, I., Didier, D., Bugmann, P., Bongard, O., and Wyss, M. (1994). Right atrial thrombosis associated with central venous catheters in children with cancer. *Med Pediatr Oncol* 22(1)**,** 53-57. doi: 10.1002/mpo.2950220110.

Yan, H.B., and Li, Y.M. (2020). Atrial thrombus as a complication of SLE and APS in an 8-year-old child. *Pediatr Rheumatol Online J* 18(1)**,** 90. doi: 10.1186/s12969-020-00484-z.

Yang, J.Y., Williams, S., Brandão, L.R., and Chan, A.K. (2010). Neonatal and childhood right atrial thrombosis: recognition and a risk-stratified treatment approach. *Blood Coagul Fibrinolysis* 21(4)**,** 301-307. doi: 10.1097/MBC.0b013e3283333c7c.

Zharkov, P., Ershov, N., Pshonkin, A., and Fedorova, D. (2017). Secondary antithrombotic prophylaxis in children with right atrium thrombosis. *Research and Practice in Thrombosis and Haemostasis* 1**,** 1183. doi: 10.1002/rth2.12012.
